# Alzheimer’s Precision Neurology: Epigenetics of Cytochrome P450 Genes in Circulating Cell-Free DNA for Disease Prediction and Mechanism

**DOI:** 10.3390/ijms24032876

**Published:** 2023-02-02

**Authors:** Ray O. Bahado-Singh, Sangeetha Vishweswaraiah, Onur Turkoglu, Stewart F. Graham, Uppala Radhakrishna

**Affiliations:** 1 Department of Obstetrics and Gynecology, Oakland University-William Beaumont School of Medicine, Royal Oak, MI 48309, USA; 2 Corewell Health William Beaumont University Hospital, Royal Oak, MI 48073, USA

**Keywords:** cytochrome P450, CYP, DNA methylation, cell-free DNA, Alzheimer’s disease

## Abstract

Precision neurology combines high-throughput technologies and statistical modeling to identify novel disease pathways and predictive biomarkers in Alzheimer’s disease (AD). Brain cytochrome P450 (CYP) genes are major regulators of cholesterol, sex hormone, and xenobiotic metabolism, and they could play important roles in neurodegenerative disorders. Increasing evidence suggests that epigenetic factors contribute to AD development. We evaluated cytosine (‘CpG’)-based DNA methylation changes in AD using circulating cell-free DNA (cfDNA), to which neuronal cells are known to contribute. We investigated CYP-based mechanisms for AD pathogenesis and epigenetic biomarkers for disease detection. We performed a case–control study using 25 patients with AD and 23 cognitively healthy controls using the cfDNA of CYP genes. We performed a logistic regression analysis using the MetaboAnalyst software computer program and a molecular pathway analysis based on epigenetically altered CYP genes using the Cytoscape program. We identified 130 significantly (false discovery rate correction q-value < 0.05) differentially methylated CpG sites within the CYP genes. The top two differentially methylated genes identified were *CYP51A1* and *CYP2S1*. The significant molecular pathways that were perturbed in AD cfDNA were (i) androgen and estrogen biosynthesis and metabolism, (ii) C21 steroid hormone biosynthesis and metabolism, and (iii) arachidonic acid metabolism. Existing evidence suggests a potential role of each of these biochemical pathways in AD pathogenesis. Next, we randomly divided the study group into discovery and validation sub-sets, each consisting of patients with AD and control patients. Regression models for AD prediction based on CYP CpG methylation markers were developed in the discovery or training group and tested in the independent validation group. The CYP biomarkers achieved a high predictive accuracy. After a 10-fold cross-validation, the combination of cg17852385/cg23101118 + cg14355428/cg22536554 achieved an AUC (95% CI) of 0.928 (0.787~1.00), with 100% sensitivity and 92.3% specificity for AD detection in the discovery group. The performance remained high in the independent validation or test group, achieving an AUC (95% CI) of 0.942 (0.905~0.979) with a 90% sensitivity and specificity. Our findings suggest that the epigenetic modification of CYP genes may play an important role in AD pathogenesis and that circulating CYP-based cfDNA biomarkers have the potential to accurately and non-invasively detect AD.

## 1. Introduction

Alzheimer’s disease (AD) is a chronic neurodegenerative disease characterized by a progressive decline in cognition. The pathological hallmarks of AD include insoluble amyloid-beta plaques and tau deposition [1]. However, amyloid beta plaques and tau deposition may not completely explain AD pathogenesis. Circulating cell-free DNA (cfDNA), released during cell apoptosis, necrosis, and inflammation, has emerged as a potential tool for monitoring cancer phenotypes. A similar mechanism of cell death may lead to the leaking of fragmented DNA into the bloodstream from neuronal cells [2]. Indeed, increased levels of circulating cfDNA are known to correlate with worsening cognition and dementia [3]. Epigenetic variation is now believed to be a part of the causative pathway leading to AD onset [4]. For example, altered DNA methylation has been observed in the AD hippocampus in regions crucial for neural differentiation. These methylation changes have been found to be correlated with the burden of deposited hyperphosphorylated tau proteins, a hallmark of AD [5]. We have recently reported an epigenetic analysis of circulating cell-free DNA for the investigation of AD [6], and in this study, we aim to perform DNA methylation profiling using circulating cfDNA, given the minimally invasive testing involved. The human brain cytochrome P450 enzymes (CYPs) have an impact on an array of important processes in the brain, for example, the metabolism of endogenous molecules, neurotoxins, and drugs [7,8]. CYPs, therefore, impact learning and memory to a significant degree, and they are now being considered potential therapeutic targets in neurodegenerative diseases [9].

The human CYP family consists of 57 genes, most of which encode for enzymes [10]. These enzymes are involved in fatty acid, cholesterol, bile acid, and drug metabolism [10,11], suggesting a link with AD pathogenesis [12,13]. Studies have begun to investigate the relationship of CYPs in AD and other dementias using urine, plasma, cerebrospinal fluid (CSF), and brain tissue [14,15,16]. Most of these are targeted studies and/or have focused on genomics [17,18] and not the DNA methylome.

Alzheimer’s precision neurology encompasses the use of “omics”, high-throughput technologies, and powerful computational and statistical approaches to identify novel system dysfunctions and to develop accurate biomarkers for AD [4]. Studies have described the possible role of epigenetic drugs in the treatment of neurodegenerative disease [19]. This is a promising role of inquiry; however, currently, the effectiveness and dosing regimens of such agents remain to be determined [20]. Many natural compounds, including vitamins, folate, polyphenols, and flavonoids, influence or induce DNA methylation changes, and they are considered to have therapeutic potential for neurodegenerative disorders [19].

Consistent with the objectives of AD precision neurology outlined above and using the example of cancer, we performed an epigenetic analysis of circulating cfDNA to investigate the potential molecular pathways of CYPs in AD pathogenesis. As noted previously, precision medicine can be conceptualized as biomarker-guided medicine [4]. Our second focus, therefore, was to also investigate the potential of minimally invasive CYP cfDNA epigenetic biomarkers for accurate AD detection.

## 2. Results

### 2.1. Study Groups

A total of 26 people with AD and 26 normal cognitively healthy controls were initially recruited. Among them, four study samples (one case and three control samples) were considered outliers (detailed elsewhere) [6]. Thus, the final analyses were based on 25 patients with AD and 23 control samples. The study groups were randomized, and their clinical and demographic details are provided in Table 1. Supplemental Table S1 provides the details of the medications that the study patients were taking.

### 2.2. Differentially Methylated Cytosines in CYP Genes

**Figure 1:**
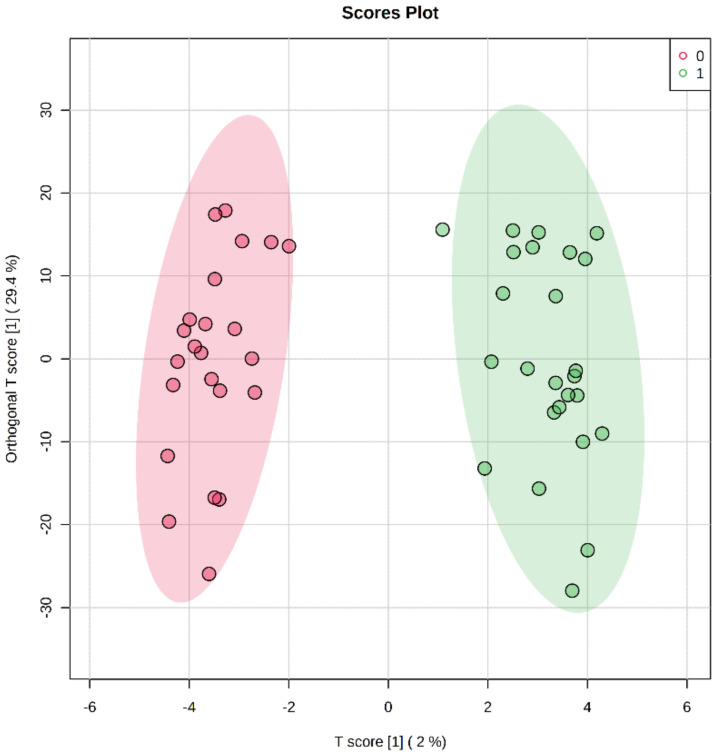
Orthogonal Partial Least Square Discriminant Analysis (OPLS-DA) scores plot of T score [1] versus orthogonal T score [1] result showing the separation of study groups. Pink nodes (0) represent cognitively healthy people, and green nodes (1) represent patients with Alzheimer’s disease.

The CpGs in the CYP genes were identified. Though there are only 57 CYP family genes [10], to achieve the most comprehensive coverage, we also included the pseudogenes and anti-sense RNA genes of CYPs. We found a total of 1044 CpGs in 66 CYP family genes. Among them, 596 CpGs were hypermethylated, and 448 CpGs were hypomethylated. However, of the 1044 CpGs, only 130 were statistically significantly differentially methylated, with 96 being hypermethylated and 34 being hypomethylated. *CYP51A1* and *CYP2S1* were the top two hypermethylated genes. The methylation status and statistical performance of the CpGs within the CYP genes are provided in Supplemental Table S2. The OPLS-DA analysis using CpG methylation indicated a clear separation between the AD and control groups (Figure 1). A cluster analysis showing the discrimination provided by the hyper- and hypo-methylated CYP CpGs is shown in Supplemental Figure S1.

### 2.3. Regression Analysis

As noted previously, the study group was divided into discovery (15 patients with AD and 13 controls) and validation (10 cases and 10 controls) sets, as detailed in Table 1. Within each group, there were no significant differences between the patients with AD and the controls in terms of age (*p*-value = 0.78), sex (*p*-value = 0.65), or BMI (*p*-value = 0.25). In the discovery/test group analysis, a predictive algorithm using CpG markers, namely, cg17852385/cg23101118 and cg14355428/cg22536554, achieved an AUC (95% CI) of 0.97 (0.95~0.99), with 100% sensitivity and 92.3% specificity (Table 2). After a 10-fold cross-validation, the performance fell slightly, resulting in an AUC (95% CI) of 0.92 (0.78~1.00) with 100% sensitivity and 92.3% specificity. Using this same predictive algorithm, an AUC (95% CI) of 0.94 (0.90~0.97) with both a sensitivity and specificity of 90% (Table 3) was achieved in the independent validation or test group. Overall, multiple predictive models each achieved a high diagnostic performance and AUC (0.915–0.941) in the independent validation/test group (Table 3).

### 2.4. Enrichment Analysis

**Figure 2:**
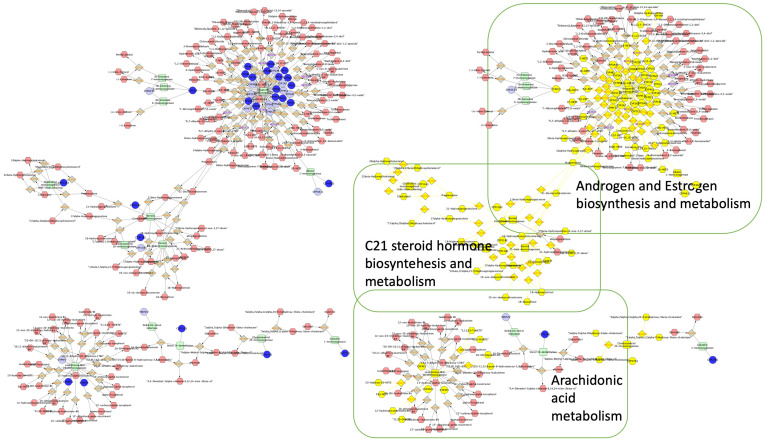
Gene–enzyme–metabolite network involving significant CpG methylations (*p* < 0.05). Blue nodes indicate the genes that were found to be significant in patients with Alzheimer’s disease. Yellow nodes indicate the genes, compounds, and enzymes involved in a particular pathway.

As noted earlier, we performed an enrichment pathway enrichment analysis to help elucidate the roles of the CYP genes in AD using circulating cfDNA. Three CYP-related pathways were found to be epigenetically perturbed in AD, namely, (i) androgen and estrogen biosynthesis and metabolism, (ii) C21 steroid hormone biosynthesis and metabolism, and (iii) arachidonic acid metabolism. These pathways are depicted in Figure 2. As explained in the Discussion Section, emerging evidence appears to validate the important roles of these CYP pathways and mechanisms in AD pathogenesis.

## 3. Discussion

CYP genes that are localized to the brain perform important functions, including arachidonic acid, sex hormone, and neurotransmitter synthesis and metabolism [21]. Emerging evidence, as noted in the Introduction Section [14,15,16], indicates a possible role of CYP enzymes in the pathogenesis of AD, a brain disorder. Consistent with the objectives of Alzheimer’s precision neurology, we performed an epigenomic analysis of CYP genes using circulating cfDNA for the minimally invasive prediction of AD and to investigate AD pathogenesis. To the best of our knowledge, there is currently very little if any data on the epigenetics of cfDNA in dementias other than AD dementia. There is limited research but significant interest in the therapeutic potential of epigenetic modifiers in AD [22]. However, the epigenetic effects of the currently administered therapeutic agents, such as cholinesterase, known to improve cognitive function in patients with AD [23], are poorly understood.

We focused on DNA methylation, as it is an important transcription regulatory mechanism of the CYP family of genes [24]. A total of 130 significant CpG loci were found to be differentially methylated in the CYP genes in AD. Given the urgent need for the development of biomarkers to detect and track the evolution of brain disorders [4], we evaluated putative CYP epigenetic biomarkers. A high diagnostic accuracy (AUC ≥ 0.90) for AD detection was achieved in an independent validation group. For example, in the independent validation group, the combination of cg17852385/cg23101118 and cg14355428/cg22536554 achieved an AUC (95% CI) of 0.94 (0.90~0.97), with the sensitivity and specificity each = 90% for AD detection. This level of diagnostic accuracy was achieved using seven different CpG methylation-based algorithms (Table 3). A limitation of conventional “big-data” analytics is the focus on statistical associations, such as diagnostic accuracy, with only limited qualitative or mechanistic data to improve the understanding of AD physiology [4]. We therefore also investigated the CYP-related mechanisms in AD pathophysiology. The top two differentially methylated loci in the CYP genes (adjusted *p*-value < 0.01) were cg01689657 (*CYP51A1*) and cg04291888 (*CYP2S1*). cg01689657 is on the TSS1500 of *CYP51A1*, and cg04291888 is on the TSS200 of *CYP2S1*. Both sites are located in the promoter region, where methylation changes are thought to be the most impactful on gene transcription. These CpG loci were hypermethylated, suggesting the possible downregulation of gene expressions. *CYP51A1* has been reported to participate in brain cholesterol biosynthesis and catabolism [25], and the *CYP2S1* gene catalyzes many reactions, including the synthesis of cholesterol and other steroids, other lipids, arachidonic acid, and drug metabolism. The downstream products of CYP2S1 arachidonic acid metabolism, e.g., epoxyeicosatrienoic acids (EETs), may play a role in angiogenesis and in the growth of tumors in humans [26]. CYP2S1′s role in the brain and particularly in AD needs to be characterized.

A pathway analysis was used to investigate the plausible role of CYPs in AD development. We identified three molecular pathways that were significantly altered in AD. Of these, the top two were sex hormones, i.e., (i) androgen and estrogen biosynthesis and metabolism and (ii) C21-steroid hormone biosynthesis and metabolism.

It is believed that the marked reduction in sex steroid hormone levels due to aging is an important risk factor for the development of AD [27]. Indeed, age and the female sex are the two strongest epidemiologic risk factors for this disorder [28]. Both estrogen and androgens are involved in the regulation of key processes in AD pathogenesis, such as β-amyloid and tau pathology, neuroinflammation, neurogenesis, memory loss, and mitochondrial impairment [27,28,29]. The cytochrome genes that were found to undergo significant epigenetic alterations in AD and that are related to these pathways were the following: *CYP1A1*, *CYP2A7*, *CYP2A13*, *CYP2C9*, *CYP2D6*, *CYP2J2*, *CYP2S1*, *CYP3A43*, *CYP4A22*, *CYP4B1*, *CYP4Z1*, *CYP11A1*, *CYP11B2*, *CYP19A1*, and *CYP21A2*. Each of these genes was reported to have pharmacological properties related to the metabolism of anti-dementia drugs [30]. (iii) Arachidonic acid metabolism: Arachidonic acid (ARA) is a polyunsaturated fatty acid belonging to the ω-6 series. ARA is released by cytosolic phospholipase A2, and the inhibition of ARA counteracts the effects of β-amyloid pathogenicity and the effects on cognition. Free ARA is involved in synaptic function, neuroinflammation, and tau hyperphosphorylation, indicating its possible role in AD pathogenesis when expressed abnormally [31,32]. The differentially methylated genes identified in our study that are known to be involved in ARA metabolism were *CYP4F3*, *CYP4F22*, *CYP27A1*, and *CYP51A1.* Among them, *CYP4F3*, *CYP27A1*, and *CYP51A1* are involved in the phase 1 reaction (oxidation, reduction, and hydrolysis) of anti-dementia drug metabolism [33]. The association of these three identified pathways in our analyses appears to provide further support for the role of CYPs in AD pathogenesis.

The limitations of this study: This study was performed using a relatively small sample size. Despite this limitation, however, we found that CYP epigenetic markers strongly and statistically significantly detected AD in an independent validation group. Further, the molecular pathways found to be epigenetically dysregulated can be credibly linked to AD mechanisms based on a review of the existing literature. The second limitation is that we studied the differential methylation of CYP family genes, but we did not directly examine the biological impact of these changes, e.g., mRNA or protein expressions in the brain. This limitation is a consequence of the generally insurmountable challenge of routinely obtaining brain tissue from living subjects. We were not able to limit analyses to circulating cfDNA from the brain. These techniques are in their infancy and are not widely validated or available commercially. We did, however, use harvesting techniques that were expected to enhance the relative concentrations of cfDNA derived from neurons, as discussed in the Materials and Methods Section.

We did not compare the DNA methylation changes in the circulating cfDNA to those in the neuronal cells of the brain, and this is important work that remains to be carried out. A prior study did, however, find a significant overlap between the methylation patterns of circulating cfDNA and the relevant brain region on a biopsy in cases of epilepsy [34], giving further plausibility to the use of cfDNA analyses in neurodegenerative disorders. Extensive work is now being carried out in developing methylation atlases of human cell types, including neurons and other brain cells [35]. Based on such atlases, we hope to be able to identify the cell of origin of circulating cfDNA more robustly and to compare the methylation profile of neuronal circulating cfDNA to that of brain neurons and other cells relevant to AD. Prior studies have indicated the feasibility of determining the tissue of origin of circulating cfDNA [36], including DNA from brain tissue.

We have previously published a study on the methylation patterns of the nuclear DNA of leukocytes in AD [37]. However, the appeal of evaluating circulating DNA is that extrapolating from cancer studies suggests the potential of cfDNA, including DNA methylation changes, as a biomarker for diagnostics, prognostics, and therapeutics [38]. A ‘liquid biopsy’ for the non-invasive investigation of brain disorders promises to deliver a powerful non-invasive tool not only for disease diagnosis but also for the ongoing monitoring of progression and, most importantly, the real-time monitoring of drug effectiveness and responsiveness.

## 4. Materials and Methods

The details of the study methods, as well as the sample collection and processing procedure, have been previously published [6], and they are summarized as follows: Study group and sample processing: We collected 26 patients with AD and 26 cognitively healthy controls. After an outlier analysis, a total of 25 patients with AD and 23 controls were used in the final study (see Table 1). AD was diagnosed based on the NINCDS-ADRDA clinical and laboratory criteria [39]. A routine CSF analysis is not a standard diagnostic criterion in our institution. The protocol was approved by the Human Investigation Committee of William Beaumont Hospital, Royal Oak, MI, USA (IRB#2017-214). Written consent was obtained from the study participants or their legal representatives. In brief, whole-blood samples were collected in Streck Cell-Free DNA BCT^®^ tubes (Streck, La Vista, NE, USA) [40]. The cfDNA was extracted twice for each sample from a total of 6 mL of plasma using a QIAamp circulating nucleic acid kit (Qiagen Cat # 55114) per the manufacturer’s standardized protocol. Generally, approximately 85% of circulating cfDNA originates from leukocytes and erythrocyte progenitors, with~2% from neurons [41]. The kit stabilizes and significantly suppresses the destruction of leukocytes and erythrocytes. This has the effect of eliminating or significantly suppressing the contributions of hematopoietic cells to the cfDNA pool. In addition, hemolyzed specimens were not used in this analysis. The overall consequence of this would be to substantially enhance the concentrations of cfDNA from other sources, such as neurons. The cfDNA was bisulfite, converted using a Zymo EZ DNA Methylation kit (Zymo, Irvine, CA, USA) [42]. The methylation assay was performed using Illumina Infinium MethylationEPIC BeadChip arrays per the manufacturer’s protocol (Illumina, Inc., San Diego, CA, USA).

### 4.1. Data Analysis

The data analysis focused on the epigenetic modification of the CpGs of CYP genes only. The data analysis was performed using R version 4.1.1. Raw EPIC array data were processed using the package “minfi”. Noob normalization was used to normalize the signal. Outliers were detected by considering the probe values that did not pass the detection threshold, and sex-chromosome-related CpGs were removed from further consideration [43]. To establish differentially methylated cytosines, the “limma” package was used. The data were adjusted for clinical and demographic covariates, and we used a false discovery rate (FDR) correction (q < 0.05) as the significance threshold. Raw beta values were used to perform an Orthogonal Partial Least Square Discriminant Analysis (OPLS-DA) and to generate a heatmap using MetaboAnalyst (v 5.0) [44]. All data, including individual CpGs, were normalized to the sum and auto-scaled before the OPLS-DA analysis [45]. OPLS-DA was performed using CpGs to assess the separation between the two groups. Models were cross-validated using permutation testing (2000 iterations). A heatmap was generated using the top 25 statistically significant CpGs when patients with AD were compared to controls.

### 4.2. Regression Analysis

The CpG beta values were expressed as ratios (e.g., CpG1/CpG2) in an attempt to increase the predictive performance of the algorithms. The CpG2/CpG1 ratio was not, however, simultaneously considered in the predictive algorithm to prevent overfitting. The prediction models were cross-validated using permutation testing (10X cross-validation) to determine whether the observed separation found in the representative score plots was statistically significant. To validate the regression models and minimize overfitting, the entire dataset was randomly split into two groups, a discovery set (15 patients with AD vs. 13 controls) and an independent test or validation set (10 patients with AD vs. 10 controls). Allocation was conducted such that there were no significant differences in demographic variables or any other potentially confounding variables between the two groups.

The demographic and clinical variables, including age, BMI, gender, history of diabetes, hypertension, diabetes, stroke, and clinical scoring systems (geriatric depression score, SLUMS, CLOX, Clinical Dementia Rating, and MMSE), were compared between the patients with AD and the controls and within discovery and validation sets, using a Student’s *t*-test or χ2 test as appropriate.

Using the discovery group, the top 100 most discriminatory CpG ß value ratios were generated by using MetaboAnalyst 5.0 [44]. A logistic regression analysis was performed using CpG ß value ratios with a stepwise variable selection method, including the Least Absolute Shrinkage and Selection Operator (LASSO) [46], to generate the diagnostic models for AD. A k-fold cross-validation (CV) technique was employed to ensure that the logistic regression models were robust [47].

The average of the 10-fold CV’s performance was used to determine the performance of the detection models developed using the discovery and training sets. The diagnostic accuracy of the top models obtained from the regression analysis in the training or model development group was then tested in the independent validation set. To determine the performance of each logistic regression model, the area under the receiver operating curve (AUC) and 95% confidence interval (CI) were calculated, as well as sensitivity and specificity values. SPSS 28.0 software was utilized to determine the thresholds via the Youden Index in order to achieve the optimal sensitivity and specificity. The model performance in both the development and test groups is provided in the Results Section.

### 4.3. Enrichment Analysis

Cytoscape software (version 3.8.2) with the Metscape tool plug was utilized to perform compound, reaction, enzyme, and gene interaction networks [48]. The CpGs that were statistically significantly (*p* < 0.05) different between the cases and the controls were included to build a gene network. Metscape can identify highly connected network components and perform network-based gene-set analyses, allowing us to compare the enriched subnetworks in the patients with AD with those in the controls.

## 5. Conclusions

To the best of the authors’ knowledge, this study is the first of its kind to use circulating cfDNA in subjects with AD in order to explore the relationship between DNA methylation in CYP genes and AD mechanisms and prediction. We identified significant methylation changes in the CpGs of two CYP genes (*CYP51A1* and *CYP2S1)* that are involved in regulating processes, such as cholesterol metabolism, neurotoxicity, and drug metabolism, each of which appears to be important in AD. These markers can be utilized to detect the disease for further confirmation. This study should be considered a proof-of-concept, and a further larger study group is needed to provide more insight into the CYP epigenetic mechanisms in AD and to confirm the findings reported in this study.

## Figures and Tables

**Table 1:** Clinical demographics and parameters comparison in discovery and validation groups.

Clinical Factor	Discovery Set			Validation Set		
	Controls (*n* = 13)	Cases (*n* = 15)	*p*-Value	Controls (*n* = 10)	Cases (*n* = 10)	*p*-Value
Age, mean (SD)	78.0 (9.6)	82.4 (5.4)	0.09 *	79.5 (8.6)	80.9 (9.4)	0.78 *
Sex (F/M)	9/4	9/6	0.61 ^	6/4	5/5	0.65 ^
BMI, mean (SD)	25.6 (4.7)	26.0 (4.4)	0.7 *	26.5 (5.7)	27.5 (4.2)	0.25 *
Diabetes, *n*	4	4	0.65 ^	2	2	1.0 ^
Hypertension, *n*	7	11	0.28 ^	8	7	0.60 ^
History of stroke/TIA	2	3	0.8 ^	1	1	0.65 ^
Family history of Alzheimer’s disease, mean (SD)	3	7	0.07 ^	3	3	0.87 ^
Geriatric depression score, mean (SD)	2.07 (1.7)	2.35 (2.23)	0.60 *	1.3	1.7	0.29 *
SLUMS total score, mean (SD)	25 (3.39)	13.16 (6.64)	0.12 *	24.4	12.1	0.02 *
CLOX 1, mean (SD)	12.07 (1.55)	9.0 (3.39)	0.21 *	12	7.7	0.60 *
CLOX 2, mean (SD)	12.58 (2.39)	10.07 (3.68)	0.29 *	13.25	10.2	0.09 *
Clinical Dementia Rating, mean (SD)	0	1.03	0.013 *	0.05	1.0	0.075 *
MMSE score, mean (SD)	29 (0.81)	20.3 (4.79)	<0.001 *	29	20.4	0.002 *

^ Chi Square; * *t*-test.

**Table 2:** Performance of top CpG methylation logistic regression models in discovery set (15 patients with Alzheimer’s disease (AD) vs. 13 controls).

CpG Methylation Predictive Algorithms	Study Group	AUC (95% CI)	Sensitivity	Specificity
cg17852385/cg23101118 + cg14355428/cg22536554	Training/Discovery	0.974 (0.957~0.992)	100 %	92.3%
	10-fold Cross-Validation	0.928 (0.787~1.000)	100 %	92.3%
cg17852385/cg23101118 + cg22195884/cg22536554	Training/Discovery	0.972 (0.954~0.991)	100%	86.3%
	10-fold Cross-Validation	0.928 (0.787~1.000)	100 %	92.3%
cg17852385/cg23101118 + cg07014416/cg22536554	Training/Discovery	0.977 (0.963~0.991)	88.1%	92.3%
	10-fold Cross-Validation	0.913 (0.771~1.000)	86.7%	92.3%
cg07014416/cg22536554 + cg17011709/cg17852385	Training/Discovery	0.977 (0.964~0.990)	93.3%	92.3%
	10-fold Cross-Validation	0.918 (0.796~1.000)	93.3%	92.3%
cg07014416/cg22536554 + cg02604290/cg17852385	Training/Discovery	0.988 (0.978~0.997)	94.1%	100%
	10-fold Cross-Validation	0.810 (0.610~1.000)	93.3%	84.6%
cg07014416/cg22536554 + cg02604290/cg17852385 + cg01689657/cg13608716	Training/Discovery	0.991 (0.984~0.998)	94.1%	92.3%
	10-fold Cross-Validation	0.908 (0.763~1.000)	93.3%	92.3%
cg17852385/cg23101118 + cg07014416/cg22536554 + cg01689657/cg13608716	Training/Discovery	0.990 (0.982~0.997)	88.1%	92.3%
	10-fold Cross-Validation	0.908 (0.765~1.000)	86.7%	92.3%

**Table 3:** Performance of these same top predictive models in the independent validation group (10 patients with Alzheimer’s disease (AD) vs. 10 controls).

CpG Predictive Models	AUC (95% CI)	Sensitivity	Specificity
cg17852385/cg23101118 + cg14355428/cg22536554	0.942 (0.905~0.979)	90.0 %	90.0%
cg17852385/cg23101118 + cg22195884/cg22536554	0.940 (0.908~0.972)	87.8%	90.0%
cg17852385/cg23101118 + cg07014416/cg22536554	0.932 (0.898~0.966)	81.1%	90%
cg07014416/cg22536554 + cg17011709/cg17852385	0.915 (0.874~0.955)	88.1%	90.0%
cg07014416/cg22536554 + cg02604290/cg17852385	0.919 (0.876~0.961)	80.0%	90.0%
cg07014416/cg22536554 + cg02604290/cg17852385 + cg01689657/cg13608716	0.925 (0.886~0.964)	80.0%	88.0%
cg17852385/cg23101118 + cg07014416/cg22536554 + cg01689657/cg13608716	0.941 (0.911~0.972)	80.1%	89.0%

## Supplementary Materials

The following supporting information can be downloaded at https://www.mdpi.com/article/10.3390/ijms24032876/s1.
