# Supplementary figures and images for: Alzheimer’s Precision Neurology: Epigenetics of Cytochrome P450 Genes in Circulating Cell-Free DNA for Disease Prediction and Mechanism
Source: Int J Mol Sci. 2023 Feb 2;24(3):2876. doi: 10.3390/ijms24032876 (PMC9917756; doi:10.3390/ijms24032876)

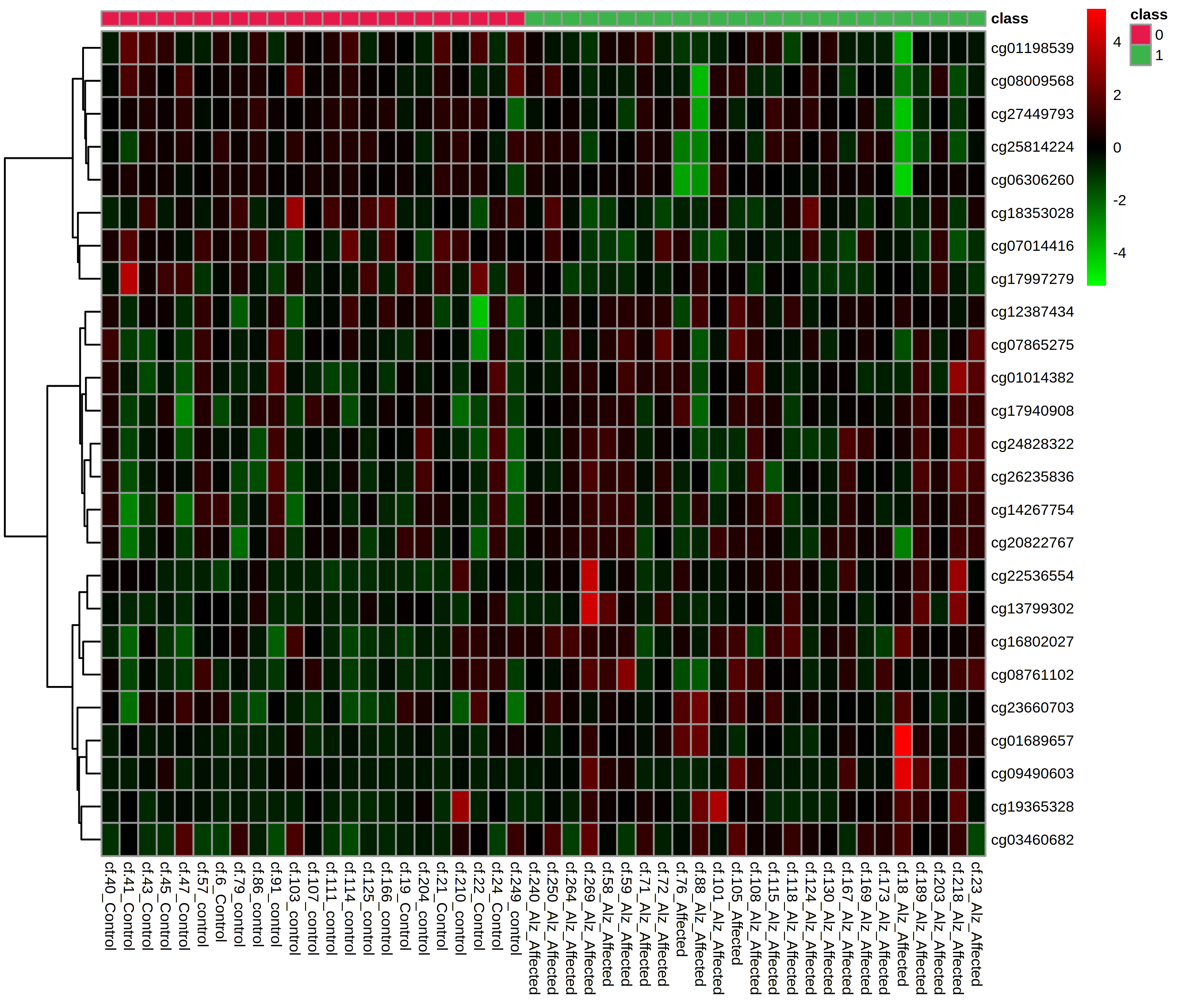

Supplement: Supplementary file 1 [file ijms-24-02876-s001.zip › 5-Supplemental Figure-1 AD cfDNA-CYP-Heatmap.png]
